# Performance of Novel Antimicrobial Protein Bg_9562 and In Silico Predictions on Its Properties with Reference to Its Antimicrobial Efficiency against *Rhizoctonia solani*

**DOI:** 10.3390/antibiotics11030363

**Published:** 2022-03-08

**Authors:** Pranathi Karnati, Rekha Gonuguntala, Kalyani M. Barbadikar, Divya Mishra, Gopaljee Jha, Vellaisamy Prakasham, Priyanka Chilumula, Hajira Shaik, Maruthi Pesari, Raman Meenakshi Sundaram, Kannan Chinnaswami

**Affiliations:** 1 Department of Pathology, Indian Institute of Rice Research, Hyderabad 500030, India; pranathi05@gmail.com (P.K.); rekha.bt89@gmail.com (R.G.); kalyaniaau@gmail.com (K.M.B.); mishradivya04@gmail.com (D.M.); vprakasam.iari@gmail.com (V.P.); cpriyanka2456@gmail.com (P.C.); hmasoodsk@gmail.com (H.S.); pesari.maruthi@gmail.com (M.P.); 2 Plant Microbe Interactions Lab, National Institute of Plant Genome Research, Aruna Asaf Ali Marg, New Delhi 110067, India; jmsgopal@gmail.com

**Keywords:** antifungal protein, sheath blight, biological control, in silico analysis, protein modeling

## Abstract

Bg_9562 is a potential broad-spectrum antifungal effector protein derived from the bacteria *Burkholderia gladioli* strain NGJ1 and is effective against *Rhizoctonia solani*, the causal agent of sheath blight in rice. In the present study, in vitro antifungal assays showed that Bg_9562 was efficient at 35 °C and 45 °C and ineffective either at high acidic pH (3.0) or alkaline pH (9.5) conditions. Compatibility studies between the native bioagents *Trichoderma asperellum* TAIK1 and *Bacillus subtilis* BIK3 indicated that Bg_9562 was compatible with the bioagents. A field study using foliar spray of the Bg_9562 protein indicated the need of formulating the protein before its application. In silico analysis predicted that Bg_9562 possess 111 amino acid residues (46 hydrophobic residues, 12 positive and 8 negative residues) with the high aliphatic index of 89.92, attributing to its thermostability with a half-life of 30 h. Bg_9562 (C_491_H_813_N_137_O_166_S_5_) possessed a protein binding potential of 1.27 kcal/mol with a better possibility of interacting and perturbing the membrane, the main target for antimicrobial proteins. The secondary structure revealed the predominance of random coils in its structure, and the best 3D model of Bg_9562 was predicted using an ab initio method with Robetta and AlphaFold 2. The predicted binding ligands were nucleic acids and zinc with confidence scores of 0.07 and 0.05, respectively. The N-terminal region (1–14 residues) and C-terminal region (101 to 111) of Bg_9562 residues were predicted to be disordered regions. Stability and binding properties of the protein from the above studies would help to encapsulate Bg_9562 using a suitable carrier to maintain efficiency and improve delivery against *Rhizoctonia solani* in the most challenging rice ecosphere.

## 1. Introduction

Management of plant pathogens using alternate methods to chemical pesticides, in the present era of consumer awareness and climate change, is a necessity more than an option. Pesticide residues are a major issue of concern in the domestic and export markets, affecting the overall income and livelihood of farmers. In this context, antagonistic microbes offer an alternative for a wide range of inputs, for such alternative eco-friendly strategies of pest and disease management [1]. Antimicrobial metabolites and proteins from the microbes offer numerous opportunities to manage the plant pathogens effectively, provided they are used appropriately [2]. These compounds are highly sensitive to environmental conditions, in particular temperature, light and moisture, and therefore need special formulations to be more viable and effective during storage, handling and in field applications [3]. There are several reports on the utilization of formulated antifungal proteins for the treatment of human infections, mostly from the prospects of drug discovery and target delivery against human pathogens [4]. Antifungal (AF) proteins in general are classified into groups based on their mode of action. The first group, which are generally amphipathic in nature, act by lysis [5], while the second group interferes with cell wall synthesis or biosynthesis of essential components such as glucan or chitin [6,7]. Use of fungal-derived antifungal and bacterial-derived antifungal proteins for the management of plant pathogens has been extensively reviewed [8].

Rice (*Oryza sativa* L.) is one of the two principal food crops in India. India is the second-largest producer, consumer and exporter, with an average production of 117.47 mT in an area of 43.79 mha [9]. The average yield losses in rice due to pests and diseases vary between 10–30% depending on the severity of the stress factors involved [10]. Among other diseases, sheath blight disease is one of the most destructive, leading to a significant yield reduction in rice [11]. Sheath blight disease of rice is caused by soil-born fungi, *Rhizoctonia solani* (RS) (Basidiomycetes; teleomorph: *Thanatephorus cucumeris*), which attacks more than 100 host plants [11]. The fungus is divided into 14 anastomosis groups (AG1 to AG13 and AGBI) among which the subgroup AG1 IA is the most common cause of disease in at least 27 families of monocots and dicots [12,13]. Currently, the disease is largely managed by the application of chemical fungicides [14]. As there is an increasing concern regarding pesticide residues in crop products and soils, non-chemical disease control is becoming increasingly popular [15], and in addition, the integrated approach with the use of biocontrol agents would reduce the chances of pathogens evolving resistance against the fungicides [16].

Recently, a prophage tail-like protein produced by the bacteria *Burkholderia gladioli* strain NGJ1 named Bg_9562 was demonstrated to have microphagous activity against RS using a Type III secretion system (T3SS; Injectisome) effector protein for entering the fungal hyphae [17]. Bg_9562 and its various orthologs were found to harbor a conserved phage_TAC_7 superfamily domain and no known toxic or lytic domain was detected in Bg_9562 and its orthologs [17]. Despite being very effective against RS, the physiochemical properties, 3D structure, mode of action as an antifungal agent and its translocation in the plant system still remain to be investigated. To address this issue, the current study is focused on identifying different properties of Bg_9562 through wet-lab experiments with RS and coupled with in silico computational approaches to predict the 3D structure of the protein for obtaining insights regarding the structural and functional characterization of the protein. We obtained the purified protein from NIPGR (National Institute of Plant Genome Research, New Delhi, India) and conducted the wet-lab in vitro assays and field studies. The physicochemical properties, transmembrane regions, and thermal stability-related residues of the protein were determined in silico and correlated with wet-lab experiments and bioactivity assays of Bg_9562 on RS. The interaction of Bg_9562 with potential native biocontrol agents *Trichoderma asperellum* TAIK1 and *Bacillus subtilis* BIK3 [18] was studied, which helps in the use of this protein along with the biocontrol agents. In summary, this study aims to identify the antimicrobial properties of Bg_9562 for its effective usage as a biocontrol agent to fight against sheath blight disease of rice.

## 2. Results

### 2.1. Antifungal Efficacy of Bg_9562 on RS

**Figure 1:**
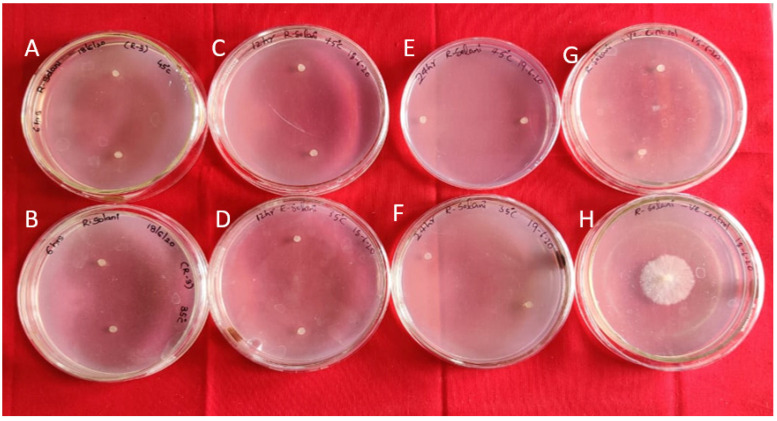
Antifungal activity of Bg_9562 protein at varied temperatures and incubation periods (*R. solani* mycelial discs): (**A**,**B**) 6 h incubation at 45 °C, 35 °C; (**C**,**D**) 12 h incubation at 45 °C, 35 °C; (**E**,**F**) 24 h incubation at 45 °C, 35 °C; (**G**) untreated protein (positive control); (**H**) *R. solani* (negative control). Observations taken after 24 h.

The purified protein was found to be suppressing the growth of RS in vitro when compared to the control. In the experiment on different treatments of protein and *R. solani* under different temperature and pH conditions, it was observed that the protein was effective at both the temperature variables (35 °C, 45 °C) and ineffective at acidic pH (3.0). It was effective in restricting mycelial growth even with increased temperatures, indicating its thermal stability (Figure 1). However, it was inactive and unable to restrict the mycelial growth at either acidic or alkaline pH but effective under neutral pH (Table S1).

### 2.2. In Vitro Compatibility of Bioagents, Trichoderma Asperellum TAIK1 and Bacillus Subtilis BIK3 with Bg_9562

**Figure 2:**
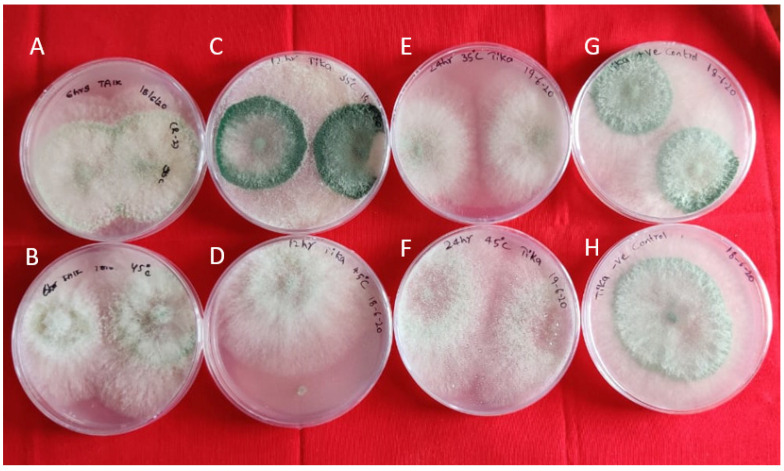
Interaction of Bg_9562 protein and TAIK1 mycelial discs at varied temperatures and incubation periods: (**A**,**B**) 6 h incubation at 45 °C, 35 °C; (**C**,**D**) 12 h incubation at 45 °C, 35 °C; (**E**,**F**) 24 h incubation at 45 °C, 35 °C; (**G**) untreated protein (positive control); (**H**) TAIK1 (negative control); no effect of protein on mycelial growth. Observations taken after 24 h.

**Figure 3:**
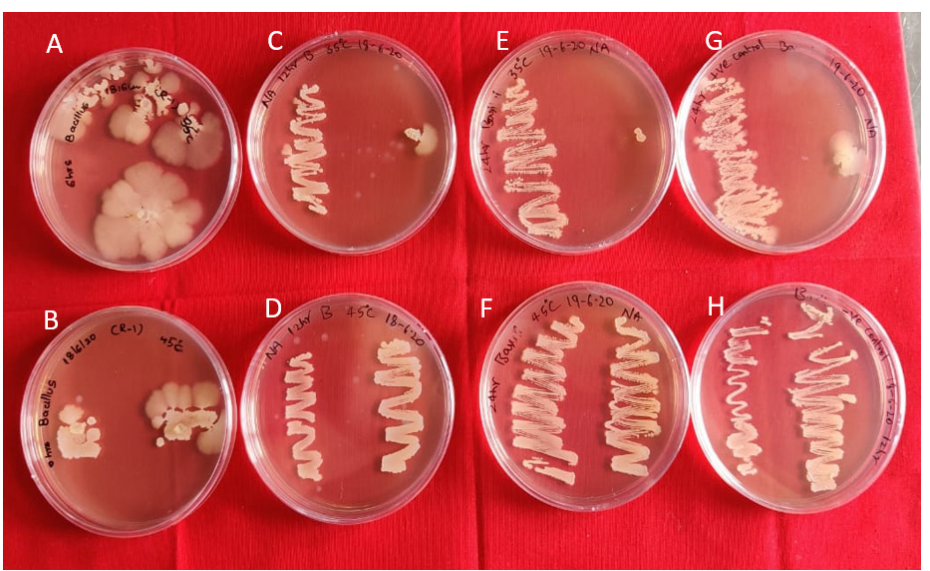
Antifungal activity of Bg_9562 protein and BIK3 colony discs at varied temperatures and incubation periods: (**A**,**B**) 6 h incubation at 45 °C, 35 °C; (**C**,**D**) 12 h incubation at 45 °C, 35 °C; (**E**,**F**) 24 h incubation at 45 °C, 35 °C; (**G**) untreated protein (positive control); (**H**) BIK3 (negative control); no effect of protein on bacterial growth. Observations taken after 24 h.

In vitro studies indicated that Bg_9562 has no adverse effect on the growth of these bioagents, at both the temperature variables (35 °C, 45 °C) at different time intervals (6 h, 12 h and 4 h), and were able to grow normally (Figure 2 and Figure 3). No inhibition in growth was observed in the antagonistic microbes tested, even after 7 days of incubation.

### 2.3. Assessment of Bg_9562 Protein Activity under Field Conditions

Data recorded in the field revealed that direct foliar treatment of plants with Bg_9562 was not efficient to manage the disease incidence, calculated as percentage disease index (PDI) in comparison to the positive control (Table S2). From the results obtained, it can be assumed that direct foliar spray may be ineffective in controlling the disease; however, formulating the protein along with compatible biocontrol agents may be effective against RS.

### 2.4. Amino Acid Composition in Bg_9562

The Bg_9562 protein sequence contains 111 amino acids (Table S3) as predicted using the Expasy-translate online application (Table S4). In silico studies on amino acid composition revealed that the Bg_9562 protein has the maximum alanine (14.4%) and minimum cysteine (0.9%) and histidine (0.9%) residues (Table S5). A complete absence of two aromatic amino acids, tryptophan and tyrosine was observed. The total number of negatively-charged residues (Asp + Glu) was 12 and the total number of positively-charged residues (Arg + Lys) was 8, while the total number of hydrophobic amino acids (Ile + Val + Lue + Phe + Cys + Met + Ala + Tyr) was 46.

### 2.5. Physiochemical Properties of Bg_9562 Determined Using ProtParam Tool

The molecular weight of Bg_9562 was predicted to be 11.5 kDa and its theoretical isoelectric point was calculated as 4.65. The total net charge was estimated as −3.75 at neutral pH, indicating that Bg_9652 has an acidic nature. Further, Bg_9562 was found to be a stable protein with the predicted instability index value of 21.19 (Table 1). The aliphatic index of Bg_9562 was estimated to be as high as 89.92. The GRAVY (grand average of hydropathicity) index of Bg_9562 was predicted to be −0.012, indicating the solubility of the protein and its hydrophilic nature. The estimated half-life of Bg_9562 was 30 h. The extinction coefficient was estimated as 0 M^−1^ cm^−1^ at 280 nm measured in water, assuming all cysteine residues are reduced and converted to cystines. Due to the absence of tryptophan residue in the Bg_9562 protein, the estimation could result in more than 10% error in the computed extinction coefficient.

### 2.6. Physiochemical Properties of Bg_9562 Determined Using Protscale

The details of other important physicochemical properties of Bg_9562 are provided in Table S6. These were predicted using the Expasy-Protscale tool (Table S4) with a default window size of 9 and values obtained were normalized so that they all fit in the range of 0 to 1.

### 2.7. Bg_9562 Properties Determined Using APD

**Figure 4:**
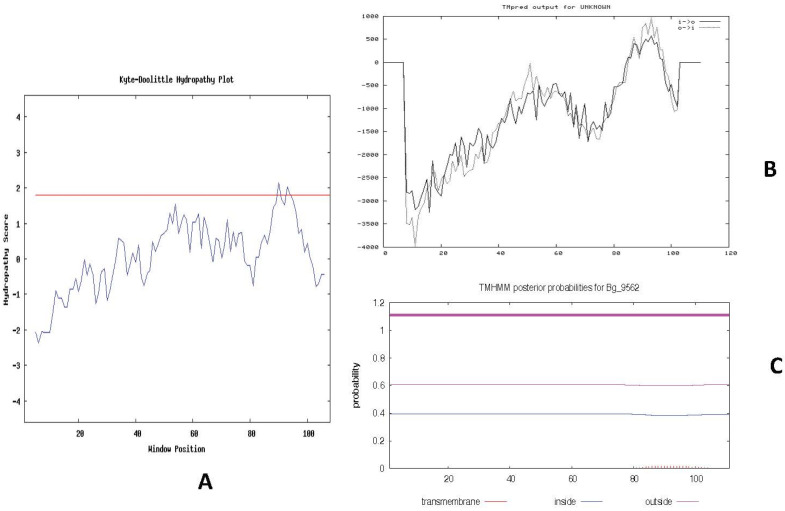
Hydropathy plot constructed for Bg_9562 protein and prediction of transmembrane region of Bg_9562. (**A**) Kyte–Doolittle hydropathy plot was constructed with 103 effective amino acids and with the window size of 9. The peaks above the red line (~1.8) in the graph indicate possible transmembrane regions in the protein. (**B**) The output of TMPred analysis shows presence of single strong transmembrane helices with 83–102 residues of Bg_9562 protein. (**C**) Prediction of transmembrane region with high probability using TMHMM software.

Using predictive tools available at Antimicrobial Peptide Database v2.34, it was found that Bg_9562 has antimicrobial activity (Table 2). The Boman index [19] estimated the protein binding potential of Bg_9562 as 1.27 kcal/mol, which indicates the potential affinity of Bg_9562 with other proteins in the cell. A higher Boman index value (>2.48) indicates that a given antimicrobial protein will be multifunctional or will play a variety of different roles within the cell due to its ability to interact with a wide range of proteins, while a low or negative Boman index value indicates a less effective antimicrobial protein. Another tool at ADP, which estimated the hydrophobicity if the amino acid contributed favorably to membrane interface partitioning of peptides, was determined by the Wimley–White scale for interfacial insertions. Interestingly, Bg_9562 displayed a high Wimley–White whole-residue hydrophobicity value of 26.18 kcal/mol, indicating that it is hydrophobic. Furthermore, it indicated that it has a better possibility of interacting and perturbing the membrane, which is the main target of action of many antimicrobial proteins. A hydropathy plot drawn using the Kyte and Doolittle scale (Table S4) (Figure 4A) predicted hydrophobic regions with a single transmembrane region in Bg_9562. Similar results were obtained with hydrophobicity (Kyte and Doolittle) estimated using the Protscale tool (Table S6), indicating the hydrophobic nature of Bg_9562. The transmembrane nature of Bg_9562 was determined using TMPred (Transmembrane Predictor) software and TMHMM-2.0 server (prediction of transmembrane helices in protein). Both the tools detected a single transmembrane region in the protein Bg_9562 between 83 to 102 amino acid positions (Figure 4B,C).

### 2.8. Propensity of Crystallization

Bg_9562 was found to crystallize with a 0.507 confidence score by the CRYSTALP2 webserver. This server generates the predictions utilizing the composition and collocation of amino acids, isoelectric point, and hydrophobicity, as estimated from the primary sequence of the given protein. Similar results were obtained with the PPCpred webserver (predictor of protein production, purification and crystallization), where the crystallization was with the propensity value of 0.23. A PPCpred predicted score of above 0.4 has the ability to crystallize the protein, thus suggesting a difficulty in crystallization of Bg_9562.

### 2.9. Sub-Program Sorting Using PSORT Tool

The possible cleavage site of Bg_9562 was estimated between 56 and 57 amino acid residues. It was noticed that no N-terminal signal peptide, no endoplasmic reticulum retention site and no peroxisomal targeting signal were present in the C-terminal region. There was no possible vacuolar targeting motif and no RNA-binding motif likewise. Similarly, there was no transport motif from the cell surface to Golgi, and no N-myristoylation pattern was found. Therefore, the κ-nearest neighbor (κ-NN) algorithm was deployed for assessing the probability of localizing at each candidate site, where κ is a predefined integer parameter. A two-fold κ-NN was employed by the PSORT tool (Protein Subcellular Localization Prediction tool), where two different κ values (κ1 < κ2) were used, localization sites were classified into two categories according to their data size, and the prediction probability for localization corresponded to the reckoned κ-data points. The results showed that κ-data points contained nuclear proteins with 47.8%, cytoplasmic proteins with 34.8%, mitochondrial proteins with 8.7% and cytoskeleton proteins with 8.7%. The Bg_9562 was predicted to be localized in the nucleus with a probability of 47.8%.

### 2.10. Secondary Structure of Bg_9562

**Figure 5:**
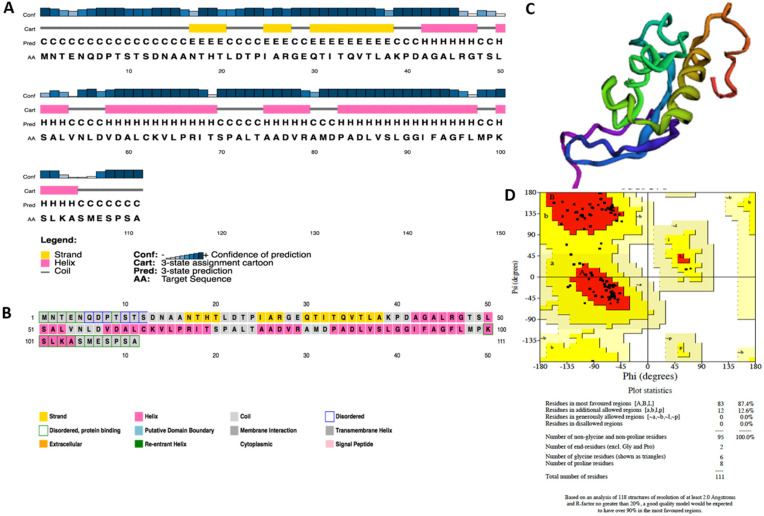
(**A**) Prediction of secondary structure of Bg_9562 using PSIPRED 4.0 work bench (**B**) Annotation grid showing the structure and function of each residue of Bg_9562 protein using PSIPRED 4.0. (**C**) 3D structure model of Bg_9562 developed using Robetta (**D**) Validation of 3D structure model using Ramachandran plot.

Secondary structure prediction of Bg_9562 using various methods of the NPS@ (Network Protein Sequence @nalysis) server estimated that there is a dominance of coiled structural components followed by helices and extended strands in Bg_9562 (Table 3). Similar results were also obtained using PSIPRED 4.0 workbench (PSIBLAST-based secondary structure PREDiction) (Figure 5A,B).

### 2.11. 3D Structure Modeling of Bg_9562

The 3D structure of Bg_9562 is not available in the Protein Data Bank (PDB) (http://www.rcsb.org/pdb, accessed on 24 January 2022) Due to the absence of a suitable structural template, homology modeling was not successfully utilized. Thus, using the comparative/homology modeling, web-based servers PHYRE 2.0 (Protein Homology/anologY Recognition Engine), HHpred, RAPTORX, (PS)^2^-v2 and SWISS MODEL generated models with less confidence, and their Verify 3D values and overall quality factor (ERRAT) were not within the accepted range (Table S7).

In order to obtain the high-quality structure of Bg_9562, ab initio and threading approaches were deployed. By using these approaches, five models were generated through LOMETs (Local Meta-Threading Server), ten models generated through SPARKS, five models from QUARK and five models from Robetta. The models generated through LOMETs and SPARKS did not qualify upon validation using Verify 3D, ERRAT and Ramachandran plot. The most appropriate approach was ab initio using Robetta and QUARK. The models generated by Robetta and QUARK were similar in Verify 3D and ERRAT, while values varied with the evaluation with Ramachandran plot (Table S7). Model four of Robetta displayed allowed values of Z-score (−7.4) and QMEAN4 (−1.42), and Verify 3D (100%) and ERRAT (98.05%) and Ramachandran plot favored regions with 87.4%. We selected this as the best model for the 3D structure of Bg_9562 (Figure 5C,D). For this predicted 3D structure, the COACH tool (consensus approach to protein–ligand binding site prediction) predicted the binding ligands as nucleic acids and zinc with lower confidence scores of 0.07 C-Score and 0.05 C-Score, respectively. C-Score, as predicted using the COACH tool, defines the confidence score of prediction and it ranges 0–1, where a higher score indicates more reliable prediction. The consensus binding residues of Bg_9562 for nucleic acids included 38, 67, 78, 90, and 91 and for that of zinc were 82 and 85 residues of Bg_9562.

**Figure 6:**
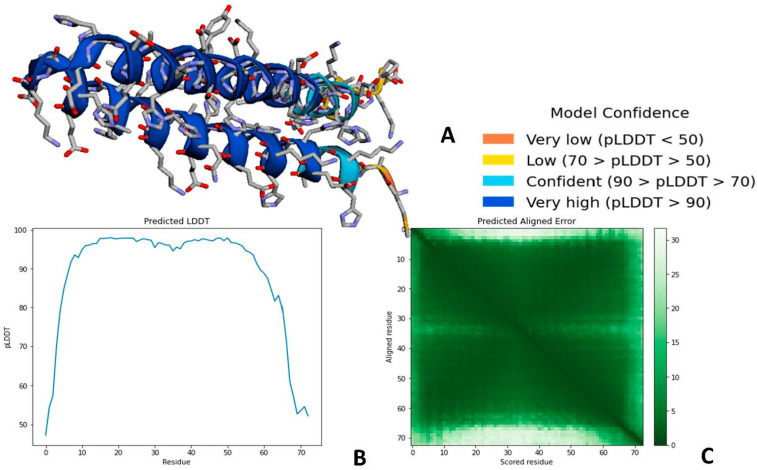
The three outputs of AlphaFold v.2.1.0. online tool. (**A**) Predicted 3D structure of Bg_9562 using AlphaFold 2. The predicted model has very high confidence levels of pLDDT > 90. (**B**) The predicted LDDT (pLDDT) plot gives best information on intra-domain confidence and it interpreted as high as pLDDT > 90. (**C**) Interactive 2D plot of Predicted Aligned Error (PAE), the dark green color at (x, y) indicated AlphaFold 2 predicted low error with well-defined relative positions from two different domains.

AlphaFold 2 also predicted a highly accurate structure of Bg_9562. The three outputs of AlphaFold 2 are represented in Figure 6. The first output is the 3D coordinates of Bg_9562 (Figure 6A). The second output is per residue confidence metric, called pLDDT, that corresponds to the model’s predicted score on the LDDT-Cα metric, which is usually in the range of 0–100. The Local Distance Difference test (LDDT) score is computed using only distances between Cα atoms in the protein model. The obtained model for Bg_9562 was found to be highly accurate, possessing the regions with pLDDT > 90. The third output was Predicted Aligned Error, which is required to assess the confidence in the domain packing and large-scale topology of the protein. The dark green color indicated low error in predicted domains and relative orientations of Bg_9562 (Figure 6C).

### 2.12. Functional Analysis of Conserved and Disordered Regions of Bg_9562

The ConSurf server provided results on the characterization of the functional regions. It indicated that the functional regions of Bg_9562 were highly conserved. The degree of conservation of the amino acid sites among 50 homologs with similar sequences to Bg_9562 was estimated. The results generated consisted of a structural representation of the protein (Figure S1a), which contains a colorimetric conservation score. Out of 1160 HMMER (homology search algorithm of ConSurf server) generated hits, 1084 were found to be unique and the calculation was performed on the 50 sequences closest to the query i.e., Bg_9562. The phylogenetic tree was constructed using a phylogeny.fr tool using default parameters and the obtained phylogenetic tree is represented in Figure S1b. The protein sequence was also subjected to motif analysis using MEME Suite 5.4.1 (Multiple Em for Motif Elicitation) and three motifs were predicted to be conserved, which are presented in Table S8. These motifs predicted using MEME were defined as recurring and fixed length patterns in the Bg_9562 sequence. These motifs were selected based on the lowest *E*-value (Expect value) of prediction. The disordered region of Bg_9562 was predicted using the PrDOS server (Protein Disorder Prediction System) and the fold index© tool. Both the tools predicted that the N-terminal region (1–14 residues) of Bg_9562 residues were disordered and the PrDOS tool predicted C-terminal residues (101 to 111) were also disordered (Figure S2a,b).

## 3. Discussion

Invasion of crops by pests and diseases has recently seen a great jump because of several factors, including high intensity and a more input-oriented system of crop cultivation [20]. Further, this increase has warranted the use of more pesticides, leading to several unwanted developments such as health issues of consumers [21] and rejection of export consignments [22]. Eco-friendly management of pests and diseases using microbes and their products has provided an alternative to the use of synthetic plant protection chemicals [23]. The search for novel antifungal agents such as secondary metabolites and antifungal proteins is of considerable interest, especially in the case of certain pathogens such as RS, where the availability of host plant resistance is not yet available [24]. Bg_9562 is a novel antifungal protein isolated from the bacteria *B. gladioli* strain NGJ and found to be very effective against RS. However, the efficient use of Bg_9562 requires a complete understanding of its physiochemical properties for possible effective formulation, identification of molecular targets, and translocation within the plant system. Antimicrobial proteins (AMPs) are currently being considered as a promising alternative for antifungal drugs. However, most of the naturally occurring and synthetically obtained AMPs have so far proven challenging to turn into therapeutic compounds due to concerns about cytotoxicity, low stability, salt sensitivity and a high cost of production. Indeed, several AMPs have displayed significant toxicity for human cell membranes (e.g., pardaxin, melittin and several cathelicidins are cytotoxic due to their membrane lytic mechanism of action [25,26,27]). In addition, it is well known that physiological salt concentrations can sometimes reduce the effectiveness of cationic AMPs. Therefore, the synthesis of short AMPs containing an appropriate mix of hydrophobic and cationic amino acids with low cytotoxicity and reduced sensitivity to salt is essentially required to design clinically useful AMPs. This strategy is being deployed in many laboratories across the globe [28,29,30]. In the absence of the crystal structure of Bg_9562 to date, we have determined its stability, structural and functional properties through wet-lab experiments and in silico computational approaches.

Bg_9562 was subjected to two different pH and temperature treatments followed by testing their effectivity against RS in vitro. These temperature and pH ranges were determined based on the conditions in which rice is grown in the country (http://www.knowledgebank.irri.org/rice-knowledge-for-india/india, accessed on 20 August 2020). Significant bioactivity of the protein at higher temperatures (45 °C) indicates the thermostability of the protein to withstand application in the late sown crop in tropical conditions, where the temperature ranges above 35 °C during most stages of the crop cycle [31]. Positive compatibility studies of Bg_9562 with native antagonistic/plant growth promoting fungi and bacteria isolated and characterized at this institute, viz., TAIK and BIK3 strains [18], would help in co-application of Bg_9562 along with the biocontrol agents for the management of ShB disease in rice. In addition to their plant protection properties, plant growth promotion by *Bacillus* and *Trichoderma* species will give an additional benefit to the plants. This combined application would thus increase the efficiency of the management of sheath blight disease, save time and energy for farmers, reduce the cost of production, and increase yield and farmers’ incomes.

A study on comparison of the amino acid sequences of the several thermophilic proteins showed that amino acid residues, viz., arginine and tyrosine, were more common, and cysteine and serine were often found in the thermophilic proteins [32]. Interestingly, Bg_9562 amino acid composition showed only slight similarity to thermophilic protein sequences, with the presence of four arginine residues and only one cysteine residue. Thus, the thermostability of Bg_9562 may be attributed to the presence of the higher aliphatic amino acids (A, I, L and V), which is similar to reported studies on various thermostable proteins [33].

Protein surface has a net charge depending on the type of amino acids and on the pH. A zero net charge due to the neutralization of charges exists at a specific pH, called the isoelectric point, mostly ranging from pH 5 to 8. Many AMPs retain antimicrobial activity at wide pH ranges. AMPs have enhanced activity at low pH due to their basic properties. This condition is related to the protonation of histidine at acidic pH that promotes electrostatic interactions with anionic surfaces. Bg_9562 was sensitive to both the pH extremities and was functional at neutral pH. Bg_9562 was predicted to possess only one histidine residue, which may have resulted in less antifungal activity in low pH conditions in our lab experiments. Bg_9562 was predicted to possess an isoelectric pI of 4.65 and net charge of −3.75 at neutral pH. The effect of pH on the antibacterial activity of AMPs varies, for example, thanatin activity at neutral pH is slightly higher than that under acidic conditions. By contrast, the activity of xylan on *E. coli*, *Listeria*, and *C. albicans* is remarkably higher at pH 5.5 than at pH 7.4 [34]. A charge at the surface enables the protein to interact with water and a zero charge protein interacts with other proteins and precipitates [35]. A class III haem peroxidase protein from leaves of *Acorus calamus*, with a molecular weight of 32 kDa, pI of 7.93, maximum activity at pH 5.5, temperature stability from 5 °C to 60 °C and optimum at 36 °C, was found to inhibit the pathogenic fungi *Fusarium moniliforme* from rice and *Macrophomina phaseolina* from black gram [36].

AMPs are generally distinguished by variations in their length, amino acid sequence and structural properties (helical, beta-sheet, turns, extended, etc.) [37,38,39]. The sequence length of Bg_9562 was found to be 111 amino acids, which is consistent with the property of antimicrobial peptides. Most of the AMPs are characterized by a short peptide length and generally consist of 12–100 amino acid residues [40]. This minimizes the probability of being degraded by bacterial proteases. In general, most of the AMPs are highly cationic, consisting of lysine (Lys) and/or arginine (Arg) residues and 50% hydrophobic amino acids. The Bg_9562 protein contains 46 hydrophobic amino acids (41%) with the maximum presence of alanine residues (14.4%). The aromatic amino acids, tyrosine and tryptophan, are completely absent and two phenylalanine residues are present in Bg_9562. In general, the presence of large aromatic residues in proteins prefers T-shaped interactions which influence the protein structure to a large extent. Individual interactions between these residues provide little stability to the structure, while the cumulative effect of their interactions contributes significantly to the conformational stability of the overall protein structure [41]. The phenylalanines have the tendency to pair with themselves—Phe–Phe pairs [42]. Tyrosine residues, on the other hand, tend to bind more with DNA and RNA and are more involved in the DNA–protein complexes [43]. The absence of tyrosine and tryptophan amino acids in the sequence of Bg_9562 might result in less affinity to be intact and might allow Bg_9562 to interact more with the sugars in the cell walls of RS. Aromatic amino acids once metabolized behave as precursors for a variety of substances in the cell [44]; however, with the absence of such aromatic amino acid residues Bg_9562 may not be able to be a precursor for the synthesis of other active compounds and may be a simple protein produced at the tail end of the metabolic pathway that is involved in the defense of the bacteria that produce it. The assessment of the secondary structure of Bg_9562 showed the presence of more random coils than the alpha helix. Random coils are one of the non-native states of proteins [45]. Non-native states of proteins are of increasing research interest because of their relevance to protein folding, translocation, and stability [46]. The 3D structure of proteins can be modeled via the comparative method (both comparative modeling and threading), which depends on known protein structures, or by ab initio, which relies on amino acid sequences. To date, comparative modeling is the most successful and accurate method, as evolutionarily related proteins usually share a similar structure (sequence identity > 30%). However, searching for homologous proteins is relatively difficult when the sequence identity is low or known as the “twilight zone”, where the sequence identity falls between 10–30%. When confronted with this problem, threading or ab initio are alternate methods to obtain the protein structure. As Bg_9562 was also categorized into the twilight zone with less sequence identity to known proteins, ab initio method-based tools were deployed, and an appropriate validated 3D model was developed using Robetta.

Most AMPs share amphipathic properties, which allow the cationic peptides to bind to negatively charged microbial membrane surfaces. In general, most of the antimicrobial peptides have a positive net charge (mostly between +2 to +4) at pH 7, which provides binding specificity to the negatively charged bacterial membranes through electrostatic interactions [47], and less than 5% of AMPs have a net negative charge. In addition, these peptides mostly have an isoelectric point close to 10 [48], which is very similar to soap or detergent. The anionic AMPs (those possessing net negative charge) have a large number of negatively charged aspartic and glutamic acid residues [49]. The net charge of Bg_9562 was predicted as −3.75 and isoelectric pI was calculated as 4.65. Bg_9562 was classified as an anionic AMP with 12 negatively charged residues (aspartic acid + glutamic acid). Anionic AMPs require zinc as a functional co-factor and the zinc complex shows stronger antibacterial activity [50]. Several of these AMPs use metal ions to form cationic salt bridges with the negatively charged components of the microbial membrane to penetrate the membrane. Anionic AMPs attach to the ribosomes or inhibit ribonuclease activity when in the cytoplasm [51]. Interestingly, zinc was also predicted as a ligand by the COACH server with a lower confidence score of 0.05 for the developed 3D model of Bg_9562. Thus, we predict that Bg_9562 may interact with zinc ions for its efficient penetration into fungal membrane. Nevertheless, further experiments are required to confirm its mode of action.

Another vital property of AMPs is hydrophobicity. Creating pores in the cytoplasmic membrane of the fungal cell requires hydrophobic residues that interact with the lipid bilayer, i.e., the hydrophobic portion [52]. This interaction results in the degradation of the cytoplasmic membrane. Low hydrophobicity may be insufficient to induce an effective interaction between these peptides and cytoplasmic membranes, which decreases antimicrobial activity. Nevertheless, the exceptionally high hydrophobicity of AMPs leads to their auto-association and inability to penetrate/enter the membranes [52]. The Bg_9562 protein documented optimal hydrophobicity of 41%, which aids in efficient interaction with fungal membranes. Interestingly, a transmembrane region from 83 to 102 amino acid residues of Bg_9562 was predicted which contributes to efficient interaction with cell membranes. The positive Boman index of Bg_9562 predicted as 1.27 kcal/mol also enhances the ability of Bg_9562 to bind to cell membrane proteins. The Boman index is essentially the average hydrophobic value measured using the Radzicka–Wolfenden scale [53]. The GRAVY index of Bg_9562 was slightly negative (−0.012), meaning that the protein is hydrophilic in nature. This is a paradox and needs to be reinvestigated. Protein solubility is variable and is affected by the number of polar and non-polar groups and their arrangement along the molecule. Generally, proteins are soluble only in strong polar solvents such as water, glycerol or formic acid, and rarely soluble in less polar solvents such as ethanol. The hydrophilic nature of Bg_9562 may indicate its high solubility in solutions. The interaction between hydrophobic groups is reported to be the most important force that maintains the tertiary structure of proteins [54]. The negative GRAVY may indicate that the tertiary structure of Bg_9562 may be less stable, leading to a flexible action upon reaching the target. However, the half-life period of Bg_9562 is very long (30 h), indicating a long persistent protein when compared to other proteins which have a half-life period ranging less than 10 h [55]. In the present study, the structure analysis of Bg_9562 indicated that it does not possess lytic domains and thus may not be toxic. In addition, Bg_9562 is reported to possess a phage tail Gp41 domain [17]. Several studies reported the use of tail proteins as novel antimicrobial peptides [56]. A novel phage tail-like bacteriocin, designated maltocin P28, was extracted from *Stentrophomonas maltophilia* and found to possess strong bactericidal activity against 38 of 81 tested *S. maltophilia* strains [57]. Similarly, the tail protein named R-type syringacin, from *Pseudomonas syringae*, was found to be highly conserved and lethal to other strains of *Pseudomonas* [58]. Interestingly, the structure prediction analysis for the intrinsically folded or disordered region in Bg_9562 sequences revealed that the N-terminal region is intrinsically disordered. Similarly, the PrDOS server estimated that both N-terminal (1–14 residues) and C-terminal (101–111 residues) regions of Bg_9562 were in disorder, while the fold index© tool indicated only the N-terminal region (1–26 residues) of Bg_9562 was disordered. The proteins that are in intrinsic disorder usually serve in the cell signaling functions and regulation. The disorder blocks in IDPs allow the simultaneous interaction with many proteins, and therefore, proteins act as multifunctional proteins in physiological conditions [59,60]. Thus, due to the presence of IDPs, Bg_9562 might be able to cope in the different physiological salt concentrations, retaining its functional ability even at higher pH concentrations. In addition, ProtParam tool analysis predicted Bg_9562 to be a stable protein with the instability index value of 21.19 and estimated half-life of >20 h, indicating the potential of Bg_9562 in enduring the process of development of different formulations, including encapsulations to be effective against sheath blight disease.

Many bacterial proteins are tethered to the inner or outer membranes via long acyl chains. These lipid anchors are covalently attached to specific N-terminal cysteines of periplasmic proteins as they emerge from the Sec machinery, forming lipoproteins, and can then be inserted into the appropriate membrane [61]. The highly hydrophobic tails of lipoproteins prevent them from crossing the periplasmic space alone, and a specialized localization of the lipoprotein (Lol) pathway is used to sort and traffic those destined for the outer membrane [61]. The PSORT tool predicted that Bg_9562 is more likely to target the nucleus of the RS, with a probability of 47.8%. Interestingly, nucleic acids present in the nucleus were predicted as ligands using the COACH server for the modeled tertiary structure of Bg_9562 with a confidence score of 0.07. It may thus be hypothesized that the mode of action of Bg_9562 is through regulation of transcription by binding to specific regions that bind to nucleic acids in *R. solani*, but further experimentations are required to confirm this hypothesis. Recent studies by [62] confirmed a similar result where a synthetic KW_4_ antifungal protein against *C. albicans* could inhibit cellular functions by binding to RNA and DNA after it has been translocated into cells, resulting in the eradication of *C. albicans*. In the same study the authors conducted several studies including fluorescence spectroscopy, scanning electron microscopy, laser-scanning confocal microscopy and gel retardation assays to confirm the mode of action of the antifungal KW_4_ protein as estimated by the in silico analysis.

## 4. Materials and Methods

### 4.1. Preparation of Pure Bg_9562 Protein

The pure form of protein Bg_9562 was obtained from National Institute of Plant Genome Research laboratory (NIPGR, Delhi, India). The laboratory procedure for protein expression and purification was described in [17]. After confirming the presence of ~13 kD band in Western blot, the purified protein Bg_9562 was utilized for all further experiments.

### 4.2. Broad-Spectrum Bioactivity Assay of Bg_9562 on Fungal and Bacterial Growth

Individual axenic cultures of RS, TAIK1 and BIK3 were grown on Potato Dextrose Agar (PDA) and Luria Broth Agar (LBA) media, respectively, and kept for incubation at room temperature. Using a cork borer, mycelial discs were collected and were dipped in 1 mL (100 µg/mL) of protein and incubated at two temperatures, viz., 35 °C, 45 °C; pH 3.0, 9.5 (*n* = 3) and at time intervals, viz., 6, 12 and 24 h along with positive (purified Bg_9562 protein at standard temperature and neutral pH) and negative controls (axenic cultures of RS, TAIK1, BIK3). After treatment, fungal/bacterial discs were washed individually in sterile distilled water and placed on fresh PDA and LBA plates and were incubated at their respective growth temperatures. Observations in the form of mycelial growth or bacterial colonies were observed after 6, 12 and 24 h in terms of mycelial lawn area (RS, TAIK1) and bacterial colonies (BIK3) observed in treated and control plates. The experiment was repeated three times.

### 4.3. Efficacy of Bg_9562 Protein under Field Conditions

To test the efficacy of Bg_9562 protein, pilot field trials were taken up in a rice variety TN1. The field trials were laid out in a Randomized Block Design with three replications and three treatments (pre-infection, post-infection and co-inoculation of RS and protein) against rice sheath blight disease at varying concentrations of the protein, viz., 2.5 ppm, 5.0 ppm, 7.5 ppm and 10 ppm, and with a spray volume of 833 mL/T maintaining positive (Carbendazim 50% WP) and negative (untreated) controls (Table S9).

### 4.4. Sequence Retrieval

Nucleotide sequence of the *Bg_9562* gene of *B. gladioli* strain NGJ1 was retrieved from the NCBI database using accession code KX620741. It was then translated to polypeptide sequence using the online tool Expasy-translate [63] and it was used for further in silico analysis in the study. The computational approaches utilized in every step of the in silico study are described briefly as a flowchart (Figure S3). Various online software/servers deployed at each step in the in silico analysis are listed and their URL details are given in Table S4.

### 4.5. Prediction of Physiochemical Properties of Bg_9562 Protein

The amino acid composition and various other physiochemical properties of Bg_9652, including theoretical pI, molecular weight, bulkiness, polarity, aliphatic index and instability index were estimated using the online tools Expasy-ProtParam [63] and Protscale [63] that are available for free use in the public domain. The hydrophobicity plot of the contiguous amino acid residues of Bg_9562 was determined by constructing the Kyte–Doolittle hydropathy graph [64]. Other parameters, i.e., Wimley–White whole-residue hydrophobicity of the peptide, protein binding potential (Boman index) and antimicrobial potential were calculated using predictive online tools of the Antimicrobial Peptide Database v.2.34 (APD2; http://aps.unmc.edu/AP/main.php, accessed on 31 August 2020) [65]. The Boman index is an estimate of protein-binding potential, calculated on the basis of cyclohexane-to-water partition coefficient of the respective amino acid side chains divided by the total number of amino acid residues within the peptide [19]. Wimley–White whole-residue hydrophobicity of the peptide (the sum of whole-residue free energy of transfer of peptide from water to phosphatidylcholine interface) calculates the protein hydrophobicity, and the higher its value the more likely the protein is to be hydrophobic [66].

### 4.6. Prediction of Functional Properties of Bg_9562 Protein

The motifs in the Bg_9562 protein were predicted using the default parameters in the MEME Suite 5.4.1 (Multiple Em for Motif Elicitation) [67]. MEME represents motifs as position-dependent letter–probability matrices that describe the probability of each possible letter at each position in the pattern. The prediction of transmembrane-spanning regions and their orientation in the protein Bg_9562 was performed using TMPred online software. The algorithm is based on the statistical analysis of TMbase, a database of naturally occurring transmembrane proteins [68]. Another tool that predicts topology of membrane proteins is based on a hidden Markov model; TMHMM-2.0 server was employed [69]. The property of localization of Bg_9562 within the cells was predicted using the PSORT program [70].

Online database CRYSTALP2 [71] was used to predict the propensity of the protein to crystallize. Either fully or partially disordered proteins have little tendency to crystallize. The sequence-based prediction of the propensity for production of diffraction-quality crystals, production of crystals, purification and production of the protein material was predicted using the online database PPCpred [72]. A ConSurf server was employed to identify the conserved and biologically important residues in the amino acid sequences of Bg_9562 [73]. The degree to which an amino acid position is evolutionarily conserved (i.e., its evolutionary rate) is strongly dependent on its structural and functional importance. Thus, conservation analysis of positions among members from the same family can often reveal the importance of each position protein structure or function. In ConSurf, evolutionary rate is estimated based on the evolutionary relatedness between the protein and its homologues and considering the similarity between amino acids as reflected in the substitution matrix methods. The multiple alignment was built using MAFT and UNIREF90 was used for the collection of homologs using HMMER homology search algorithm of the ConSurf database. These generated sequences that were closest to the given query, i.e., Bg_9562 were selected for phylogenetic tree representation. The phylogenetic tree was constructed using Pylogeny.fr, an online database tool [74].

### 4.7. Secondary Structure Prediction

The secondary structure of Bg_9562 protein was predicted using PSIPRED 4.0 workbench [75] and in addition, the secondary structure of the protein was also predicted employing NPS@ server. The Network Protein Sequence @nalysis (NPS@) is an interactive webserver dedicated to protein sequence analysis [76]. Secondary structure prediction using NPS@ server was performed with various methods, viz., DSC [77], HNN [78], PHD [79], PREDATOR [80], MLRC [81] SIMPA96 [82] and secondary consensus [83] keeping default parameters for 4 state predictions and keeping output width = 70.

### 4.8. 3D Structure Prediction

To obtain a high-quality model of Bg_9562 protein, its tertiary structure was modeled using in silico methods including homology modeling, threading and ab initio. For comparative homology modeling, web-based server HHPred [84] was used and the best alignment suggested by HHpred that is typically collected using the PSIBLAST program was used. The atomic coordinates built on this target template alignment were generated using MODELLER v. 9.2 [85]. Another web-based server, Phyre2, was used, which uses the hidden Markov method to generate alignments of a submitted protein sequence against proteins with published structures [86]. The resulting alignments were then used to produce homology-based models of the query sequence to predict its three-dimensional structure. RaptorX uses a non-linear scoring function to combine homologous information with structural information for a given template–sequence alignment. It uses NEFF to adjust the relative importance of homology and structural information. RaptorX uses a combination of RaptorX-Boost and Raptor-X MSA to build 3D models for a target–template alignment [87]. SWISS MODEL was used for predicting and analyzing homology-based 3D protein structure [88]. Web-based threading servers, LOMETS (which builds 3D models by collecting high-scoring target-to-template alignments from locally installed threading programs FFAS3D, HHsearch, MUSTER, pGenTHREADER, PPAS, PRC, PROSPECT2, SP3 and SPARKS-X) [89] and I-TASSER that generates 3D models based on the hierarchical method for protein structure and function prediction were employed [90]. Further, SPARKSX, that constructs 3D models based on the application of probabilistic-based matching between predicted primary structural properties of the query and corresponding native properties of templates was also used in the study [91]. For proteins such as Bg_9562, which has very little homology with known templates, another method, ab initio, was deployed. Ab initio is a de novo protein structure prediction that builds 3D models of proteins from primary structures in the presence or absence of homologs to the given query protein. Robetta by Baker’s lab, whose protein structure prediction is continuously evaluated using CAMEO, was deployed for ab initio method [92] and another tool was QUARK [93]. As the existing methods fall far short of atomic accuracy, especially when no homologous template is available, another most powerful tool based on neural network-based model, AlphaFold 2 [94], was utilized to build highly accurate model of Bg_9562.

AlphaFold 2 on Google Colab’s Notebook [95] was used to model Bg_9562. This Colab notebook allows easy prediction of protein using a slightly simplified version of AlphaFold v.2.1.0. Colab notebooks are coupled to a special sequence search program, MMSeqs2, thus making this technology more powerful.

### 4.9. 3D Structure Validation

The 3D structure validation of modeled protein was carried out by PROSA, QMEAN4 and Ramachandran webservers. Using PROCHECK [96] plot to visualize backbone torsional angles, ψ and φ of residues in protein structures were drawn. It is a universal way to calculate the number of residues in favored, allowed and outlier regions in the given protein. PROSA webserver is a frequently employed tool in the validation of protein structures obtained from X-ray analysis, NMR spectroscopy and theoretical calculations. QMEAN4 is a linear combination of four statistical potential terms. It predicts a global lDDT score in the range (0,1) and the value calculated by the webserver is transformed into a Z-score [97]. After selecting the best evaluated 3D structure of Bg_9562, ligands binding to it were predicted using the COACH server [98]. COACH is consensus approach to protein–ligand binding site prediction. When the given structure is subjected to COACH server, it predicts complementary ligand binding sites using combined multiple prediction results of algorithms from TM-SITE, S-SITE, COFACTOR, and FINDSITE [98].

### 4.10. Prediction of Disordered Regions in Bg_9562

The disordered regions in the protein have immense importance in governing the structure and function of a protein. Disordered regions of Bg_9562 were identified using Protein Disorder Prediction System (PrDOS) server [99]. PrDOS predicts natively disordered regions of protein from its amino acid sequence and returns the disordered probability of each residue as prediction result. The default parameters with 5% false positive rate were used while executing the disordered regions of Bg_9562. Another simple versatile tool, fold index©, was deployed for estimating the disordered regions in Bg_9562. Fold index© predicts if the given protein intrinsically unfolded by implementing the algorithm of Uversky and co-workers based on the average residue hydrophobicity and net charge of the sequence [100].

## 5. Conclusions

The current study of the in silico analysis of physiochemical properties coupled with wet-lab experiments of Bg_9562 help in better understanding of the protein and its functional characterization, further facilitating in the identification of fungal–protein interactions and membrane and nucleic acid interactions associated with the broad-spectrum antifungal activity of the protein. Furthermore, considering its broad-spectrum antifungal activity, the protein may be potentially useful in biotechnological applications to control fungal diseases. According to our results, Bg_9562 is a stable protein with a single transmembrane region, possesses the right amount of aliphatic index and hydropathicity index values, and is essentially nontoxic to be used as an antifungal drug. In short, bioinformatics analysis would be helpful to choose the functional and targeted segments of the protein for preparations of specific formulations for controlling sheath blight disease of rice.

## Figures and Tables

**Table 1:** List of physiochemical properties of Bg_9562 protein using ProtParam tool.

S. No	Parameters	Values/Scores
1	Number of amino acids	111
2	Molecular weight	11,451.97
3	Theoretical isoelectric point	4.65
4	Total number of negatively-charged residues (Asp + Glu)	12
5	Total number of positively-charged residues (Arg + Lys)	8
6	Total number of hydrophobic amino acids (Ile + Val + Lue + Phe + Cys + Met + Ala + Tyr)	46
7	Total number of atoms	1612
8	Formula	C_491_H_813_N_137_O_166_S_5_
9	Instability index	21.19
10	Aliphatic index	89.92
11	Grand average of hydropathicity index (GRAVY)	−0.012
12	Estimated half-life (mammalian reticulocytes, in vitro)	30 h
13	Estimated half-life (yeast, in vitro)	>20 h
14	Estimated half-life (*Escherichia coli*, in vivo)	>10 h
15	Extinction coefficient	0 M^−1^ cm^−1^

**Table 2:** List of various parameters of Bg_9562 predicted using Antimicrobial Peptide Database (APD)-based prediction.

S. No.	Parameter	Values
1	Protein binding potential (Boman index)	1.27 kcal/mol
2	The Wimley–White whole-residue hydrophobicity of the peptide	26.18 kcal/mol
3	APD defined total hydrophobic ratio of protein	41%
4	Total net charge	−3.75
5	Antimicrobial activity	Yes

**Table 3:** Secondary structure of Bg_9562 predicted using NPS@ server.

Secondary Structure	DSC *	HNN *	MLRC *	PHD *	PREDATOR	Secondary Consensus
Alpha helix	55.86%	27.3%	26.13%	34.23%	22.52%	36.04%
Extended strand	3.6%	7.21%	9.91%	16.22%	3.6%	9.91%
Random coil	40.54%	65.77%	63.96%	49.55%	73.87%	52.25%

* DSC—Discrimination of Protein Secondary structure Class. * HNN—Hierarchical Neural Network method. * MLRC—Multivariate Linear Regression Combination. * PHD—Prediction Heidelberg secondary structure prediction method.

## Data Availability

Not applicable.

## Supplementary Materials

The following supporting information can be downloaded at: https://www.mdpi.com/article/10.3390/antibiotics11030363/s1, Figure S1: Functional properties of Bg_9562 predicted using Consurf server. Figure S2: Prediction of disordered region of Bg_9562 using PrDOS and fold index© tools. Figure S3: Flow diagram describing the in silico tools used in the study. Table S1: Effect of Bg_9562 protein on *R.solani* mycelial growth. Table S2: Protein efficiency check under field conditions during foliar treatment in cultivar TN1. Table S3: Amino acid sequence of Bg_9562 protein. Table S4: List of online tools and their URLs utilized in the study. Table S5: Amino acid composition of Bg_9562 protein. Table S6: Minimum and maximum values of physiochemical properties of Bg_9562 protein predicted using Protscale. Table S7: Models of Bg_9562 calculated by various methods/servers and their validation results. Table S8: Motifs predicted in Bg_9562 using MEME software. Table S9: Evaluation of efficacy of Bg_9562 protein molecule against sheath blight of rice (*Cv*. TN-1) under field conditions.
