# Supplementary material for: Performance of Novel Antimicrobial Protein Bg_9562 and In Silico Predictions on Its Properties with Reference to Its Antimicrobial Efficiency against Rhizoctonia solani
Source: Antibiotics (Basel). 2022 Mar 8;11(3):363. doi: 10.3390/antibiotics11030363 (PMC8944631; doi:10.3390/antibiotics11030363)
Supplement: Supplementary file 1 [file antibiotics-11-00363-s001.zip › antibiotics-1591149-supplementary.pdf]

antibiotics

Supplementary Materials

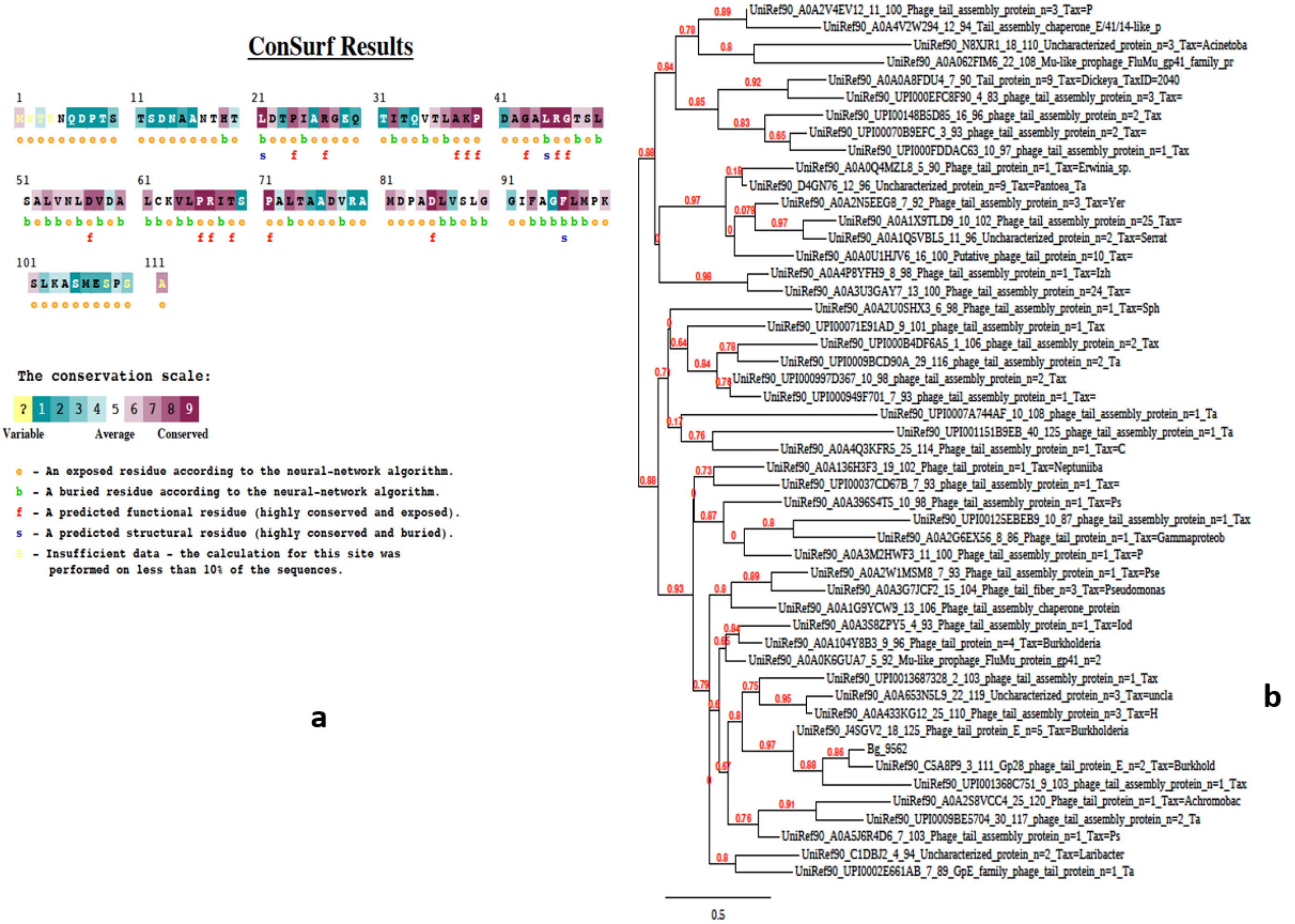

Figure S1. Functional properties of Bg_9562 predicted using Consurf server. a) Prediction of functional residues in Bg_9562 using Consurf server b) Representation of 50 molecular sequences homologous to Bg_9562 in the form of phylogenetic tree using Phylogeny.fr tool

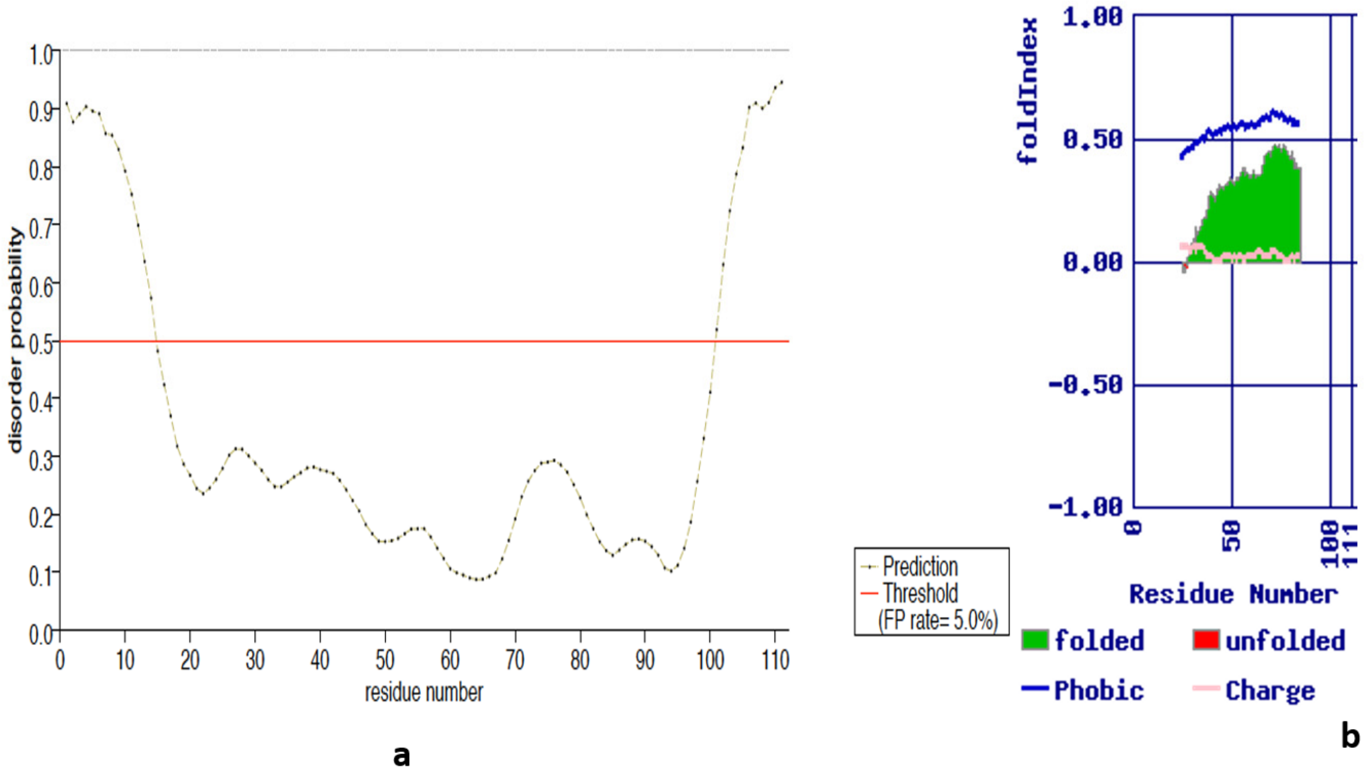

Figure S2. Prediction of disordered region of Bg_9562 using PrDOS and fold index© tools. a) Prediction of disorder regions of Bg_9562 prediction using PrDOS server. 1-14 residues at N-terminal end and 101-111 residues at C-terminal end were predicted disordered regions in Bg_9562. b) Prediction of disordered regions of Bg_9562 using fold index tool. 1-26 residues of N-terminal region were predicted as disordered region in Bg_9562. The other output parameters of fold index include unfoldability 0.202 and charge: 0.036, Phobic: 0.499.

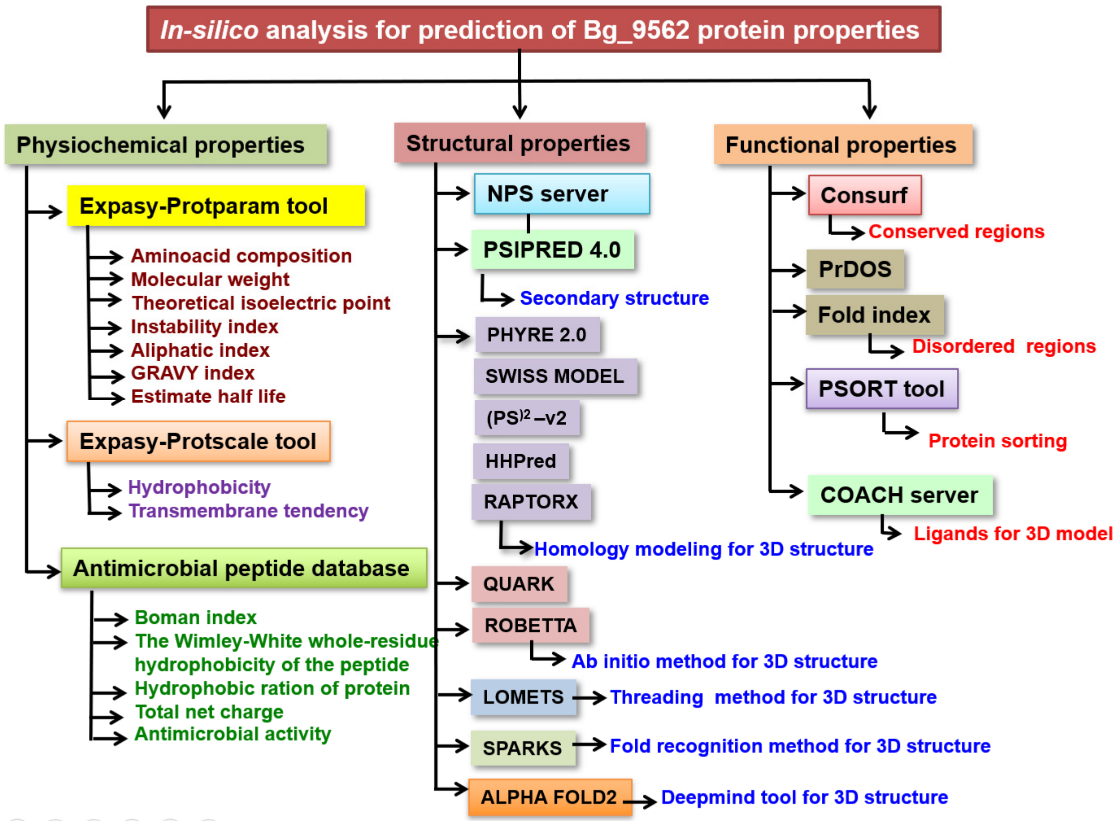

Figure S3. Flow diagram describing the *in-silico* tools used in the study.

Table S1. Effect of Bg_9562 protein on *R.solani* mycelial growth.

Parameter		Incubation period								
		6 hrs of incubation in the treated protein and mycelial growth in PDA plates observed after			12 hrs of incubation in the protein and mycelial growth observed after			24 hr incubation of incubation in the in the protein and mycelial growth observed after		
		12 hrs	24 hrs	48 hrs	12 hrs	24 hrs	48 hrs	12 hrs	24 hrs	48 hrs
pH	3.0	+	+	+	+	+	+	+	+	+
	9.5	+	+	+	+	+	+	+	+	+
Temperature	35^0 C	–	–	–	–	–	–	–	–	–
	45^0 C	–	–	–	–	–	–	–	–	–
	-ve control (*R.solani*)	+	+	+	+	+	+	+	+	+
	+ve control (*R.solani* + protein)	+	+	+	+	+	+	+	+	+

"+" - Mycelial growth is seen; " –" Mycelial growth is not seenTable S2: Protein efficiency check under field conditions in cultivar TN1.

Table S2. Protein efficiency check under field conditions in cultivar TN1.

S. No	Treatments	Concentration	R1	R2	R3	Mean (Observed)	Mean (Treated)	% increase in disease over standard check
1	Pre inoculation spray	2.5 ppm	96.1	94.7	98.6	96.4	79.5	53.19
2	Co-inoculation spray	2.5 ppm	92.3	95.3	90.2	92.6	74.3	49.96
3	Post inoculation spray	2.5 ppm	90.1	92.4	98.3	93.6	76.0	51.07
4	Pre inoculation spray	5.0 ppm	86.4	88.5	84.3	86.4	68.4	45.59
5	Co-inoculation spray	5.0 ppm	78.3	83.2	81.1	80.9	64.1	41.93
6	Post inoculation spray	5.0 ppm	84.8	82.3	81.9	83.0	65.6	43.33
7	Pre inoculation spray	7.5 ppm	81.8	80.0	78.9	80.2	63.6	41.50
8	Co-inoculation spray	7.5 ppm	78.3	77.9	78.3	78.2	62.1	40.11
9	Post inoculation spray	7.5 ppm	79.0	77.0	78.9	78.3	62.2	40.21
10	Pre inoculation spray	10.0 ppm	74.0	79.1	76.5	76.5	61.0	39.04
11	Co-inoculation spray	10.0 ppm	78.8	79.7	77.1	78.5	62.4	40.37
12	Post inoculation spray	10.0 ppm	76.6	79.4	77.2	77.7	61.8	39.83
13	Carbendazim 50% WP	1 g/l	41.4	34.7	33.9	36.7	37.2	0.09
14	Untreated control	-	91.1	94.7	92.6	92.8	74.5	50.06

Table S3. Amino acid sequence of Bg_9562 protein.

**Bg_9562**	MNTENQDPTSTSDNAANTHTLDTPIARGEQTITQVTLAKPDAGALRGTSLSALVNLDVDALCKVLPRITS PALTAADVRAMDPADLVSLGGIFAGFLMPKSLKASMESPSA

Table S4. List of online tools and their URLs utilized in the study.

Online tool/server/software	URL
Expasy-Translate	http://us.expasy.org/tools/protparam.html/
Expasy-Protscale	http://web.expasy.org/ protscale
Expasy-ProtParam	http://web.expasy.org/ proparam
Kyte-Doolittle Online Hydropathy plot	http://gcat.davidson.edu/DGPB /kd/kyte-doolittle
ADP v2.34	http://aps. unmc.edu/AP/main.php
TMPred	http://www.ch.embnet.org/software/TMPRED_form.html
TMHMM	http://www.cbs.dtu.dk/services/TMHMM/
MEME	https://meme-suite.org
PSORT	http://psort.nibb.ac.jp
CRYSTALP2	http://biomine.cs.vcu.edu/servers/CRYSTALP2
PPCpred	http://biomine.cs.vcu.edu/servers/PPCpred
ConSurf	https://consurf.tau.ac.il
Phylogeny.fr	http://www.phylogeny.fr/index.cgi
PSIPRED 4.0	http://bioinf.cs.ucl.ac.uk/psipred
NPS server	https://npsa-prabi.ibcp.fr/cgi-bin/npsa_automat.pl?page=/NPSA/npsa_server.html

Phyre 2	http://www.sbg.bio.ic.ac.uk/phyre2
RaptorX	http://raptorx.uchicago.edu/StructurePrediction/predict/
SWISS MODEL	https://swissmodel. expasy.org
LOMETS	http://zhanglab.ccmb.med.umich.edu/ LOMETS
I-TASSER	http://zhanglab.ccmb.med.umich.edu/I-TASSER
SPARKS	http://sparks-lab.org/yueyang/server/SPARKS-X/
QUARK	http://zhanglab.ccmb.med. umich.edu/QUARK
Robetta	https://robetta.bakerlab.org
Alpha fold2 on Googles Colab's Notebook	https://colab.research.google.com/github/deepmind/alphafold/blob/main/notebooks/AlphaFold.ipynb
PROCHECK	https://servicesn.mbi.ucla.edu/ PROCHECK
QMEAN 4	https://swissmodel.expasy.org/qmean
PROSA	https://prosa.services.came.sbg.ac.at/prosa.php
COACH server	https://zhanglab.ccmb.med.umich.edu/COACH
PrDOS	http://prdos.hgc.jp/cgi-bin/top.cgi
Fold index©	http://bioportal.weizmann.ac.il/fldbin/findex

Table S5. Amino acid composition in Bg_9562 protein.

**Amino acid**	**Composition in Bg_9562 protein**	**% of amino acid composition**
Ala (A)	16	14.4
Arg (R)	4	3.6
Asn (N)	5	4.5
Asp (D)	9	8.1
Cys (C)	1	0.9
Gln (Q)	3	2.7
Glu (E)	3	2.7
Gly (G)	6	5.4
His (H)	1	0.9
Ile (I)	4	3.6
Leu (L)	13	11.7
Lys (K)	4	3.6
Met (M)	4	3.6
Phe (F)	2	1.8
Pro (P)	8	7.2
Ser (S)	10	9.0
Thr (T)	12	10.8
Trp (W)	0	0
Tyr (Y)	0	0
Val (V)	6	5.4
Pyl (O)	0	0
Sec (U)	0	0

Table S6. Minimum and maximum values of physiochemical properties of Bg_9562 protein predicted using *Protscale.*

S.No.	Property	Minimum value	Maximum value
1	Bulkiness	0.437	0.802
2	Polarity	0.222	0.676
3	Refractivity	0.208	0.460
4	Hydrophobicity (Kyte and Doolittle)	0.237	0.738
5	Transmembrane tendency	0.353	0.828
6	Buried residues	0.197	0.792
7	Accessible residues	0.356	0.697
8	Ration of hetero end/side	0.059	0.389
9	Average flexibility	0.426	0.778
10	Relative mutability	0.329	0.810

Table S7. Models of Bg_9562 with different methods and their evaluation results.

Model/Tool	Approach	PROSA Z-score	Qmean4	Verify 3D	ERRAT	Outlier	Allowed region	Favored region
QUARK-1	Ab initio	-4.92	-9.47	100%	73.78	6.3%	33.7%	53.7%
QUARK-2	Ab initio	-5.37	-7.43	99.1%	87.37	2.1%	33.7%	60.0%
QUARK-3	Ab initio	-2.97	-9.68	100%	75.72%	4.2%	36.8%	53.7%
QUARK-4	Ab initio	-3.49	-9.41	100%	72.81%	4.2%	30.5%	56.8%
QUARK-5	Ab initio	-4.32	-9.3	100%	91.2%	6.3%	29.5%	60.0%
Robetta-1	Ab initio	-6.87	-2.25	89.19%	93.93%	0%	12.6%	86.3%
Robetta-2	Ab initio	-5.92	-1.33	100%	97%	1.1%	10.5%	87.4%
Robetta-3	Ab initio	-6.3	-1.15	100%	98.01%	0%	18.9%	80.0%
**Robetta-4**	**Ab initio**	**-7.4**	**-1.42**	**100%**	**98.05%**	**0%**	**12.6%**	**87.4%**
Robetta-5	Ab initio	-5.44	-1.16	100%	96.03%	1.1%	6.3%	92.6%
LOMETS-1	Threading	-4.23	-2.83	68.4%	38%	1.1%	9.5%	89.5%
LOMETS-2	Threading	-3.32	-4.46	37.84%	44.89%	2.1%	11.6%	86.3%
LOMETS-3	Threading	-3.05	-3.74	87. 39%	60	1.1%	9.5%	88.4%
LOMETS-4	Threading	-3.6	-.4.11	56.76%	46.5	1.1%	5.3%	91.6%
LOMETS-5	Threading	-2.21	-5.08	36.94%	39.1	1.1%	8.4%	89.5%
SPARKS-1	Fold recognition	-4	-6.99	96.4%	56.4	0%	16.8%	75.8%
SPARKS-2	Fold recognition	-2.59	-7.96	75.68%	33.9	0%	20.0%	76.8%
SPARKS-3	Fold recognition	-2.66	-6.61	76.58%	35.9	0%	14.7%	82.1%
SPARKS-4	Fold recognition	-1.87	-6.85	83.78%	25.2	2.1%	14.7%	81.1%
SPARKS-5	Fold recognition	-3.75	-9.39	23.42%	30.09	2.1%	26.3%	67.4%
SPARKS-6	Fold recognition	-1.13	-6.20	50.45%	21.6	1.1%	20.0%	76.8%
SPARKS-7	Fold recognition	-2.93	-6.21	85.59%	29.12	1.1%	14.7%	80.0%
SPARKS-8	Fold recognition	-4.81	-4.97	83.74%	46.6	0%	18.9%	78.9%
SPARKS-9	Fold recognition	-3.18	-6.65	50.4%	63	1.1%	12.6%	82.1%
SPARKS-10	Fold recognition	-3.04	-6.30	72.70%	41.5	1.1%	13.7%	83.2%
PHYRE 2.0	Homology modeling	-0.96	-2.15	0%		0%	4.8%	95.2%
$(PS)^2$-v2	Homology modeling	-3.61	-5.86	36.94%	45.54	3.2%	9.5%	86.3%
SWISS-model-1	Homology modeling	-2.4	-2.93	61.9%	100	0%	9.1%	87.9%
SWISS-model-2	Homology modeling	-1.08	-1.76	0%	100	0%	15.6%	84.4%

RAPTORX	Homology modeling	-0.26	-4.71	12.61%	63.52	2.1%	4.2%	91.6%
HHpred-1	Homology modeling	-2.94	-1.07	0	41.6%	0%	0%	100%

Table S8. Motifs predicted in Bg_9562 primary sequence.

Motif logo	p-value	Start	E-value	Site count	Width
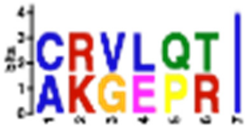	3.87e-9	62	7.8e+001	2	7
	9.69e-8	26			
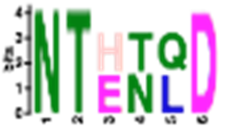	1.93e-8	2	3.0e+001	2	6
	7.17e-7	17			
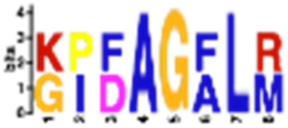	3.64e-10	91	1.8e+002	2	8
	3.04e-8	39			

Table S9. Evaluation of efficacy of Bg_9562 protein molecule against sheath blight of rice (*Cv*. TN-1) under field conditions at ICAR-IIRR, Hyderabad.

S. No	Treatments	Concentration	Area/Plants	Spray volume/Tr	*Spray volume/Con
1	Pre inoculation spray	2.5 ppm	30Plants (10X3R)	833 ml	2500ml
2	Co-inoculation spray	2.5 ppm	30Plants	833 ml	
3	Post inoculation spray	2.5 ppm	30 Plants	833 ml	
4	Pre inoculation spray	5.0 ppm	30Plants (10X3R)	833 ml	2500ml
5	Co-inoculation spray	5.0 ppm	30Plants	833 ml	
6	Post inoculation spray	5.0 ppm	30 Plants	833 ml	
7	Pre inoculation spray	7.5 ppm	30Plants (10X3R)	833 ml	2500ml
8	Co-inoculation spray	7.5 ppm	30Plants	833 ml	
9	Post inoculation spray	7.5 ppm	30 Plants	833 ml	
10	Pre inoculation spray	10.0 ppm	30Plants (10X3R)	833 ml	2500ml
11	Co-inoculation spray	10.0 ppm	30Plants	833 ml	
12	Post inoculation spray	10.0 ppm	30 Plants	833 ml	
13	Carbendazim 50% WP	1 g/l			
14	Untreated control	-			

*Total amount of protein molecule required (including handling wastage at field) is **~200mg.**
